# The guiding value of hybrid resting full-cycle ratio and fractional flow reserve strategy for percutaneous coronary intervention in a Chinese real-world cohort with non-ST elevation acute coronary syndrome

**DOI:** 10.3389/fcvm.2022.991161

**Published:** 2022-09-07

**Authors:** Yumeng Lei, Shuaiyong Zhang, Mengyao Li, Jiawang Wang, Yunfei Wang, Lei Zhao, Wei Yan, Ming Chen, Yanjie Su, Jing Yu, Na Yu, Tongjun Dong, Xufen Cao, Liqiu Yan

**Affiliations:** Department of Cardiology, Cangzhou Central Hospital, Hebei Medical University, Cangzhou, China

**Keywords:** resting full-cycle ratio, fractional flow reserve, non-ST elevation acute coronary syndromes, percutaneous coronary intervention, hybrid RFR-FFR strategy

## Abstract

**Objective:**

The study aimed to assess the correlation and agreement between resting full-cycle ratio (RFR) and fractional flow reserve (FFR), and evaluate the guiding value of a hybrid RFR-FFR strategy for percutaneous coronary intervention (PCI) in a Chinese real-world cohort with non-ST elevation acute coronary syndrome (NSTE-ACS).

**Materials and methods:**

A total of 109 patients with NSTE-ACS (149 diseased vessels), who underwent an invasive physiological assessment in Cangzhou Central Hospital, Hebei Medical University, were prospectively enrolled from September 2021 to May 2022. FFR ≤ 0.80 was used as the gold standard for coronary artery functional ischemia. We utilized the Pearson correlation and Bland-Altman analysis to assess the correlation and agreement between RFR and FFR. The diagnostic value of RFR predicting FFR ≤ 0.80 was evaluated in accordance with the receiver operating characteristic (ROC) curve. The hybrid RFR-FFR strategy, which was established according to determining the “gray zone” of RFR (FFR was further assessed using vasodilators only for diseased vessels in the “gray zone”), needed to afford over 95% global agreement with the FFR-only strategy.

**Results:**

Resting full-cycle ratio was significantly linearly linked with FFR (*R*^2^ = 0.636, *P* < 0.001). The accuracy, specificity, and sensitivity for RFR ≤ 0.89 predicting FFR ≤ 0.80 were 81.2, 70.8, and 86.1%, respectively. The area under the ROC curve for RFR predicting FFR ≤ 0.80 was 0.881 (*P* < 0.001), and the cutoff value was 0.90. The “gray zone” of RFR was 0.85–0.93. The positive and negative predictive values of the hybrid RFR-FFR strategy were 0.95 and 0.93, respectively. The hybrid RFR-FFR strategy exhibited an agreement of 96.0% with FFR and obviated the need for a vasodilator by 60.4%.

**Conclusion:**

Resting full-cycle ratio and FFR have high correlation and consistency. The hybrid RFR-FFR strategy highlights considerably enhanced agreement with the FFR-only strategy, whilst making the requirement of vasodilator administration less than a half.

## Introduction

Fractional flow reserve (FFR) is the gold standard for functional assessment of the severity of coronary artery stenosis (1). Many clinical studies and guidelines have emphasised that FFR-guided percutaneous coronary intervention (PCI) can bring more clinical benefits to patients with stable coronary artery disease (CAD) (2–7). However, guidelines do not advocate FFR for patients with acute coronary syndrome (ACS) (8). Furthermore, FFR assessment needs vasodilators (such as adenosine) to reach the maximal hyperemia, and there are some disadvantages such as prolonging the operation time, increasing the examination cost, and possibly causing side effects associated with vasodilator administration (9–11), so adoption of FFR in clinical practice is still low. Previous studies have shown that the use rate of FFR is only 6–8% worldwide (12), while that in China is only 1% (13).

Recently, non-hyperemic pressure ratios (NHPRs), such as the resting ratio of distal mean pressure and aortic mean pressure (Pd/Pa) and instantaneous wave-free ratio (iFR), have attracted more and more attention because they do not need vasodilators. Two international multicenter large-scale randomized controlled studies have shown that the coronary revascularization guided by iFR was not inferior to FFR in patients with stable angina pectoris and ACS (14, 15). The resting full-cycle ratio (RFR) is one of the newly developed non-hyperemic pressure-derived indicators, which is the lowest value of the Pd/Pa of coronary stenosis over the whole cardiac cycle (16). Studies have shown that RFR is highly consistent with iFR and can be used to identify functionally significant stenosis (16, 17). However, there has not been any report about RFR in Chinese population. This study aimed to evaluate the correlation and consistency between RFR and FFR and to evaluate the guiding value of a hybrid RFR-FFR strategy for PCI in a Chinese real-world cohort with non-ST elevation acute coronary syndrome (NSTE-ACS).

## Materials and methods

### Study population

A total of 122 patients (165 vessels) with NSTE-ACS, who underwent invasive physiological examination of coronary artery in Cangzhou Central Hospital, Hebei Medical University from September 2021 to May 2022, were prospectively enrolled. 12 patients (15 vessels) who did not evaluate RFR and 1 patient (1 vessel) with FFR data drift were eliminated. Finally, 109 patients (149 vessels) were included in this study. Inclusion criteria of this study: (1) age ≥ 18 years; (2) met the diagnostic criteria of NSTE-ACS (18); (3) coronary angiography showed that the degree of stenosis was 30–90%, and (4) consented to coronary invasive physiological assessment. NSTE-ACS, which is divided into non-ST elevation myocardial infarction (NSTEMI) and unstable angina (UA) on the basis of cardiac biomarkers of necrosis, is defined according to 2020 ESC Guidelines as follows (8): acute chest discomfort with positive cardiac biomarkers but no persistent ST-segment elevation that may include transient ST-segment elevation, ST-segment depression, or T-wave inversions. Myocardial infarction (MI) is defined according to the reported fourth universal definition of MI (19). UA is defined as myocardial ischemia at rest or on minimal exertion in the absence of acute cardiomyocyte injury/necrosis and is subdivided into resting angina, initial angina, worsening angina, and variant angina according to appropriate clinical context (8). Exclusion criteria: (1) presence of severe bronchial asthma or intolerance to vasodilators such as adenosine; (2) atrioventricular block of degree II or above; (3) cardiogenic shock; and (4) RFR was not evaluated or there was data drift. This study has been reviewed by the ethics committee of Cangzhou Central Hospital, Hebei Medical University, and informed consent was provided by all patients.

### Physiological assessment of coronary artery

According to the current guidelines and standards, all patients underwent coronary angiography *via* the radial artery. The severity of coronary artery stenosis was visually determined by two experienced interventional cardiologists, and the need for invasive physiological evaluation was determined according to the patient’s clinical condition. Before physiological evaluation, 200°ug nitroglycerin was routinely given in the coronary artery to prevent coronary spasms, and the PressureWire™x0.014 pressure guidewire (Abbott Vascular Inc., Santa Clara, CA, United States) was placed beyond the lesion of interest. First, the resting Pd/Pa and RFR values were measured in the non-hyperemic state, Then, the disodium adenosine triphosphate was given at a dose of 167°ug/min/kg through the median elbow vein to reach the maximal hyperemia and determine the FFR value.

### Hybrid resting full-cycle ratio-fractional flow reserve strategy

To establish a hybrid RFR-FFR strategy, the “gray zone” of RFR values was identified by exploratory analysis: the upper limit value with a high negative predictive value (> 90%) to exclude lesions with FFR > 0.80 (defer RFR value) and the lower limit value with a high positive predictive value (> 90%) to identify lesions with FFR ≤ 0.80 (treatment RFR value). Furthermore, the hybrid RFR-FFR strategy needed to afford over 95% global agreement with the FFR-only strategy. Only lesions with RFR values falling within the “gray zone” would have been given adenosine and followed standard FFR assessment.

### Statistical analysis

Kolmogorov-Smirnov normality test was conducted for continuous variables. Variables with normal distribution were presented as mean ± standard deviation, and those with non-normal distribution were expressed as median and inter-quartile range (IQR). Categorical variables were presented as frequency (percentage). The correlation and agreement between RFR and FFR were analyzed by Pearson correlation and Bland–Altman test. The diagnostic value of RFR for predicting FFR ≤ 0.80 was evaluated in accordance with the receiver operating characteristic (ROC) curve. The accuracy, positive predictive value, and negative predictive value of the hybrid RFR-FFR strategy and FFR-only strategy were compared by consistency test. Bilateral *P* < 0.05 was taken as a statistically significant. All data were assessed statistically by Statistical Product and Service Solutions (SPSS) 25.0 and R version 4.2.0 (R Foundation for Statistical Computing, Vienna, Austria).

## Results

### Clinical baseline and angiographic characteristics

The average age of patients was 64.0 ± 8.6 years, including 50 female patients (45.9%). The clinical baseline features of patients are demonstrated in Table 1. The percentage of patients with hypertension, dyslipidemia, diabetes, and smoking history were 61.5, 6.4, 26.6, and 14.7%, respectively. 101 (92.7%) patients presented with UA and 8 (7.3%) patients with non-ST-segment elevation myocardial infarction (NSTEMI). The angiographic characteristics, physiological evaluation, and final treatment strategy of patients are shown in Table 2. Among them, 89 (59.7%), 21 (14.1%), and 39 (26.2%) were left anterior descending artery, left circumflex, and right coronary artery lesions, respectively, and the stenosis degree of 135 (90.6%) vessels was ≥ 70%. The median values of RFR, resting Pd/Pa and FFR were 0.93 (0.88–0.96), 0.95 (0.92–0.98), and 0.85 (0.78–0.90), respectively. 45 (30.2%) diseased vessels were finally treated with PCI, and 104 (69.8%) were treated with conservative drugs.

**TABLE 1:** Baseline clinical characteristics.

	Patients (*n* = 109)
Age, years, mean ± SD	64.0 ± 8.6
Female, n (%)	50 (45.9%)
BMI, kg/m^2^, median (IQR)	25.5 (24.3–26.8)
Hypertension, n (%)	67 (61.5%)
Dyslipidemia, n (%)	7 (6.4%)
Diabetes, n (%)	29 (26.6%)
Smoking, n (%)	16 (14.7%)
Drinking, n (%)	12 (11.0%)
Previous AMI, n (%)	1 (0.9%)
Previous PCI, n (%)	10 (9.2%)
Previous stroke, n (%)	13 (11.9%)
Atrial fibrillation, n (%)	4 (3.7%)
Peripheral vascular disease, n (%)	1 (0.9%)
Creatinine, μmol/L, median (IQR)	63.0 (52.5–71.0)
**Clinical presentation, n (%)**	
Unstable angina	101 (92.7%)
NSTEMI	8 (7.3%)

IQR, interquartile range; BMI, body mass index; AMI, acute myocardial infarction; PCI, percutaneous coronary intervention; NSTEMI, non-ST elevation myocardial infarction; RFR, resting full-cycle ratio; FFR, fractional flow reserve.

**TABLE 2:** Angiographic and physiological characteristics and treatment strategies.

	Lesions (*n* = 149)
**Clinical indication, n (%)**	
Unstable angina	137 (91.9%)
NSTEMI with culprit lesion	2 (1.3%)
NSTEMI with non-culprit lesion	10 (6.7%)
**Location of diseased vessels, n (%)**	
Left anterior descending	89 (59.7%)
Left circumflex	21 (14.1%)
Right coronary artery	39 (26.2%)
**Angiographic stenosis, n (%)**	
40–49%	1 (0.7%)
50–59%	6 (4.0%)
60–69%	7 (4.7%)
≥ 70%	135 (90.6%)
RFR, median (IQR)	0.93 (0.88–0.96)
**Results of RFR, n (%)**	
Positive (≤ 0.89)	48 (32.2%)
Negative (> 0.89)	101 (67.8%)
Resting Pd/Pa, median (IQR)	0.95 (0.92–0.98)
FFR, median (IQR)	0.85 (0.78–0.90)
**Results of FFR, n (%)**	
Positive (≤ 0.80)	48 (32.2%)
Negative (> 0.80)	101 (67.8%)
**Final treatment strategy, n (%)**	
Interventional therapy	45 (30.2%)
Medication	104 (69.8%)

IQR, interquartile range; NSTEMI, non-ST elevation myocardial infarction; RFR, resting full-cycle ratio; FFR, Fractional flow reserve; UA, unstable angina.

### Correlation and consistency analyses between resting full-cycle ratio and fractional flow reserve

**FIGURE 1:**
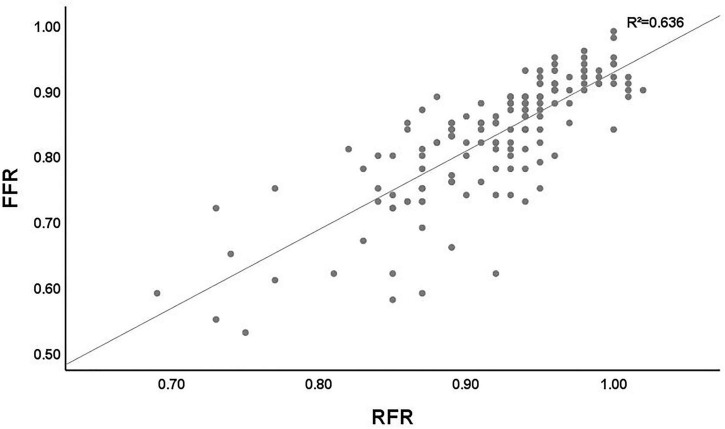
Distribution of the lesions according the RFR and FFR. RFR, resting full-cycle ratio; FFR, fractional flow reserve.

**FIGURE 2:**
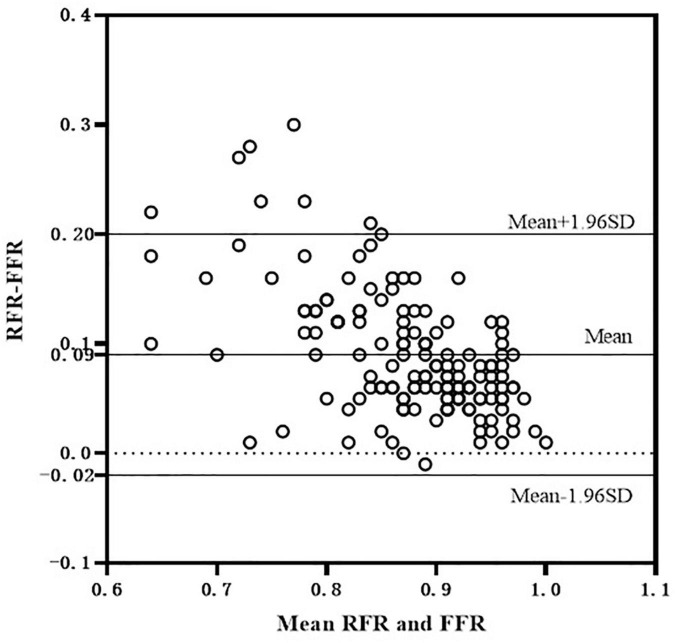
Bland-Altman consistency analysis of RFR and FFR. RFR, resting full-cycle ratio; FFR, fractional flow reserve.

The scatter plot distribution of RFR and FFR is illustrated in Figure 1. RFR and FFR were significantly linearly correlated (*R*^2^ = 0.636, *P* < 0.001). The Bland–Altman analysis showed that the average value of the difference between RFR and FFR was (0.089 ± 0.112), and the 95% confidence interval (CI) was −0.02∼0.20. There was a high degree of consistency between RFR and FFR (Figure 2).

### Diagnostic performance of resting full-cycle ratio vs. fractional flow reserve

**FIGURE 3:**
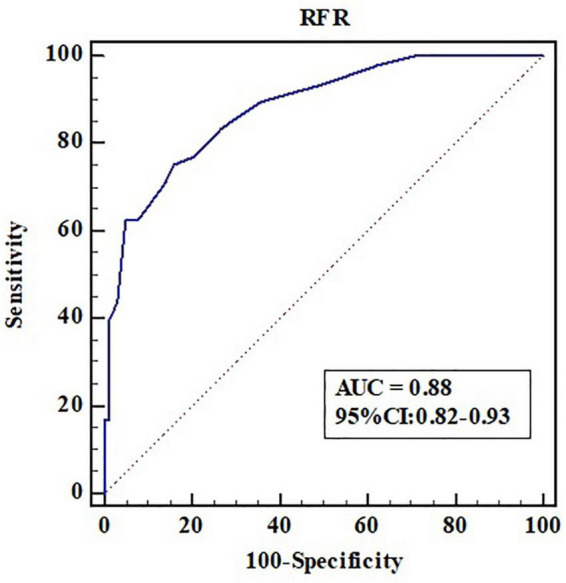
Receiver operating characteristic curve analysis of RFR in predicting FFR ≤ 0.80. AUC, area under the curve; CI, confidence interval; RFR, resting full-cycle ratio; ROC, receiver operating characteristic; FFR, fractional flow reserve.

Fractional flow reserve ≤ 0.80 was taken as the gold standard for judging functional ischemia of the coronary artery. The accuracy, sensitivity, and specificity of RFR ≤ 0.89 in diagnosing FFR ≤ 0.80 were 81.2, 70.8, and 86.1%, respectively; The positive and negative likelihood ratios were 5.11 and 0.34, respectively. The area under ROC curve (AUC) of RFR predicting FFR ≤ 0.80 was 0.88 (95% CI: 0.82–0.93, *P* < 0.001), and the cutoff value was 0.90 (Figure 3).

### Comparison between the hybrid resting full-cycle ratio-fractional flow reserve strategy and the fractional flow reserve-only strategy

**FIGURE 4:**
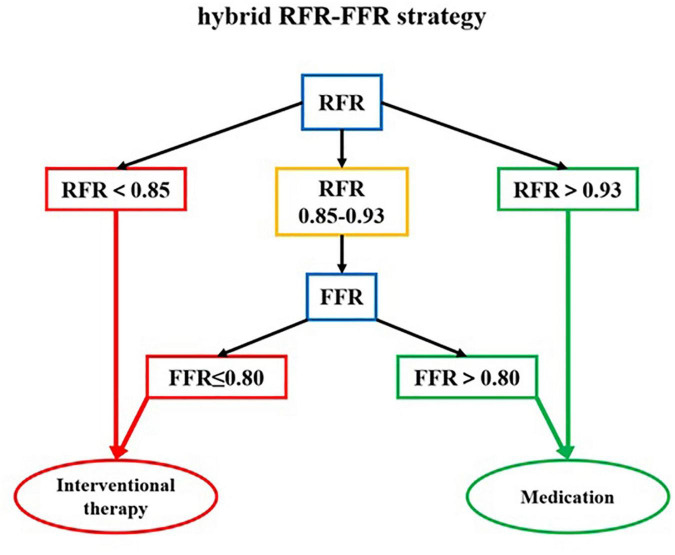
The diagnosis flow chart of the hybrid RFR-FFR strategy. RFR, resting full-cycle ratio; FFR, fractional flow reserve.

The “gray zone” of the RFR values is 0.85–0.93. When the RFR of the diseased vessel is less than 0.85, PCI is recommended. When the RFR of the diseased vessel is greater than 0.93, conservative treatment with drugs is recommended. If the RFR value falls within the “gray zone,” vasodilators are given intravenously to further evaluate the FFR, and the final treatment strategy is determined according to the FFR value. According to this hybrid strategy, when the RFR value of diseased vessels is not in the “gray zone,” adenosine or other vasodilators are not required. The diagnosis process of the hybrid RFR-FFR strategy is shown in Figure 4.

**FIGURE 5:**
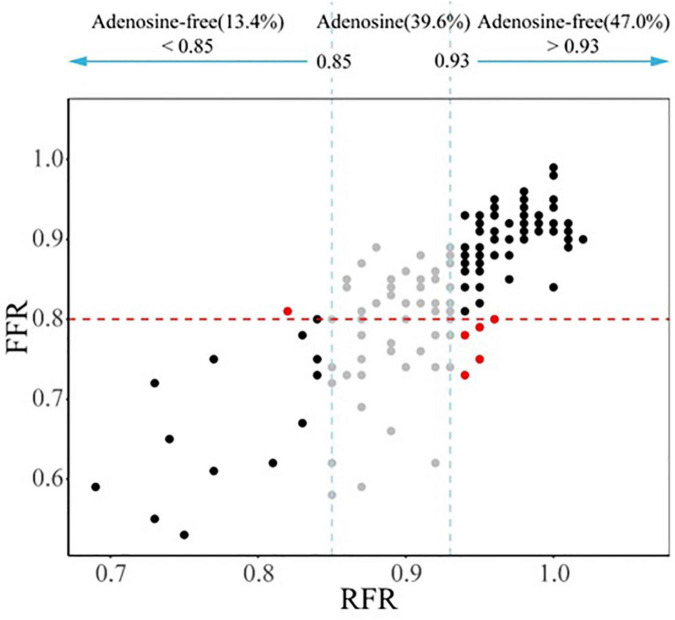
Comparison between the hybrid RFR-FFR strategy and FFR-only strategy. RFR, resting full-cycle ratio; FFR, fractional flow reserve.

The hybrid RFR-FFR strategy only misclassified 6 diseased vessels: one false positive and five false negatives. The positive predictive value of the hybrid RFR-FFR strategy was 95.0% and the negative predictive value was 93.0%. The hybrid RFR-FFR strategy exhibited an agreement of 96.0% with FFR and obviated the need for a vasodilator by 60.4% (Figure 5).

## Discussion

Previous studies have fully demonstrated that FFR-guided coronary revascularization can bring more clinical benefits to patients, reduce stent implantation, and save medical resources (20–22). The 15-year clinical follow-up results of the DEFER study showed that for patients with non-ischemic lesions (FFR > 0.75), the incidence of myocardial infarction in the PCI group was higher than that in the drug treatment group (21). FAME II study showed that FFR-guided PCI could significantly improve the quality of life of patients, reduce the onset of angina pectoris and medical costs, and significantly reduce the incidence of urgent revascularization in patients with stable CAD (3). The application of FFR in clinical practice has been unanimously recommended by revascularization guidelines for patients with stable CAD in the United States, and Europe (1, 23). However, adoption of FFR in clinical practice is still low (6–8%) (12). There are many reasons for this phenomenon, including incomplete reimbursement, lack of wide and convenient access to vasodilator drugs, challenges related to operating techniques, and lack of sufficient knowledge and correct attitude to functional assessment and guidelines (24).

In recent years, NHPRs derived from FFR, such as resting Pd/Pa, iFR, and diastolic pressure ratio (dPR), have attracted more and more attention. Since NHPRs do not need vasodilators, it is easier and faster to operate and can bring better diagnosis and treatment experience to patients. A recently published clinical study evaluated the applicability of resting Pd/Pa in guiding the revascularization of non-infarct related arteries in patients with ST-segment elevation myocardial infarction (STEMI) from the Compare-Acute trial. The results showed that resting Pd/Pa had 80.2% diagnostic accuracy in predicting FFR ≤ 0.80 immediately after PCI of non-infarct related arteries and had similar performance with FFR for predicting MI and/or revascularization of target vessels during 36 months of follow-up (25). iFR is based on a specific period of cardiac diastole (i.e., from the beginning of 25% of cardiac diastole to 5 ms before the end of diastole) when the coronary artery microcirculation waveform is in a short resting state [i.e., the wave-free period (WFP)]. ADVISE study showed that iFR and FFR had high correlation and consistency. The sensitivity, specificity, and accuracy of iFR ≤ 0.83 in diagnosing FFR ≤ 0.80 were 85, 91, and 80%, respectively (26). DEFINE-FLAIR (14) and SWEDEHEART (15) studies showed that revascularization guided by iFR was not inferior to revascularization guided by FFR with respect to the risk of major adverse cardiac events at 1°year. The procedural time and the rate of adverse procedural signs and symptoms in the iFR guiding group were shorter or lower with iFR than with FFR.

Unlike iFR, which needs to assume maximal resting blood flow and minimal microcirculatory resistance during WFP, RFR is not limited by a specific waveform-free period. VALIDATE RFR study (16) showed that only 98.1% of RFR occurred in diastole (98.5% in the left coronary artery and 93.5% in the right coronary artery). The result suggests that the resting functional indicators that only measure diastole may miss systole, especially when measuring the right coronary artery. Therefore, RFR may have greater clinical applicability. The prospective, multicenter RECOPA study (27) included 311 patients (380 lesions) with stable angina pectoris or ACS. The results showed that RFR was significantly correlated with FFR (*R*^2^ = 0.81, *P* < 0.001). The accuracy, sensitivity, and specificity of RFR ≤ 0.89 in diagnosing FFR ≤ 0.80 were 79, 76, and 80%, respectively. Another single-center “real world” clinical study from Germany further validated the results of RECOPA study (28). Based on a Chinese real-world cohort with NSTE-ACS for the first time, our study verified the significant correlation between RFR and FFR (*R*^2^ = 0.636, *P* < 0.001). The accuracy, sensitivity, and specificity of RFR ≤ 0.89 in diagnosing FFR ≤ 0.80 were 81.2, 70.8, and 86.1%, respectively, which were in line with the results of the above studies.

Although the DEFINE-FLAIR (14) and SWEDEHEART (15) studies have showed that revascularization guided by iFR was not inferior to FFR. Because of the incomplete consistency between iFR and FFR, it is often not accepted in clinical practice to simply use iFR to guide PCI. Therefore, exploring the hybrid strategy between iFR and FFR may be more conducive to the promotion of functional revascularization. Petraco et al. first discussed the guiding value of the hybrid iFR-FFR strategy for revascularization. The hybrid strategy conducted interventional treatment for the diseased vessels with iFR < 0.86, delayed revascularization for the diseased vessels with iFR > 0.93, and only further FFR evaluation for the diseased vessels with iFR in the “gray zone” (0.86–0.93). The positive and negative predictive values of the hybrid strategy were 0.92 and 0.91, respectively. While ensuring the accuracy of more than 95%, the use of adenosine was reduced by 57% (24). The RESOLVE Study, which sought to assess the diagnostic accuracy of iFR and resting Pd/Pa with FFR in a core laboratory-based multicenter collaborative study, determined different iFR thresholds necessary to achieve ≥ 90% to 99% diagnostic accuracy and demonstrated that the adenosine-free zone was inversely related to the diagnostic accuracy of the hybrid iFR-FFR strategy. There were 64.9, 28.6, and 18.0% of lesions falling within the adenosine-free zone to achieve ≥ 90, ≥ 95, and ≥ 99% diagnostic accuracy, respectively. Subsequently, the ADVISE in-practice study (29) and ADVISE II study (30) further validated the accuracy and feasibility of the hybrid iFR-FFR strategy.

Although our study demonstrated that RFR had a good ability to distinguish ischemia defined as an FFR ≤ 0.80 (AUC: 0.88, 90% CI: 0.82–0.93, *P* < 0.001), there was 18.8% discordance rate between RFR and FFR in identifying ischemia. Therefore, it may not be appropriate to simply use RFR-only strategy to guide coronary revascularization in our daily practice. It is necessary to establish an adenosine administration “gray zone” to improve the ability to identify ischemia, which seems to be the most appropriate strategy. The newly published RECOPA study explored the guiding value of the hybrid RFR-FFR strategy for revascularization in patients with CAD. The “gray zone” of RFR established in this study was 0.86–0.92. Compared with the FFR-only strategy, the positive and negative predictive values of the hybrid RFR-FFR strategy were 0.91 and 0.92 respectively, and the accuracy was 95.3%. At the same time, the use of vasodilators was reduced by 58%. Our study, for the first time, established a “gray zone” of RFR of 0.85–0.93, which was slightly different from that in the RECOPA study (0.86–0.92), in a Chinese real-world cohort with NSTE-ACS. One possible explanation for the difference lies in the fact that the two populations are not identical. The positive and negative predictive values of the hybrid RFR-FFR strategy were 0.95 and 0.93 respectively, and the accuracy was 96.0%. At the same time, the use of vasodilators was reduced by 60.4%. Unlike most previous studies, which mainly evaluated intermediate lesions, our study included a large proportion of patients with NSTE-ACS with stenosis ≥ 70%. The results of this study highlight that the hybrid RFR-FFR strategy is also feasible for NSTE-ACS with severe coronary stenosis. While ensuring the accuracy of more than 95%, it can reduce the use of vasodilators by more than a half.

In our study, there were 90.6% patients with diameter stenosis ≥ 70% and only 32.2% lesions with positive FFR, which was significantly different from the FAME trial. There may be some reasons as follows. First, the degree of stenosis in these studies was estimated by visual assessment rather than quantitative coronary angiography, which may be overestimated or underestimated in different medical centers. Second, the functional significance of lesions depends not only on the degree of stenosis, but also on the myocardial blood supply range of diseased vessels, the length and location of the lesions, and so on. Third, the proportion of lesions with positive FFR and undergoing revascularization in our study was similar to that in the multicenter real-world RECOPA study (30.2 vs. 32.7%) and significantly lower than that in the multicenter randomized controlled FAME study (61%), which may be the difference between the real-world studies and randomized controlled clinical trials.

### Limitations

This study has the following limitations. First of all, it is a single center registry study, which may limit extrapolation of the findings to other population. Second, only 8 (7.3%) patients with NSTEMI were included in our study, which is contrary to the current trend of a low percentage of patients with NSTE-ACS not having troponin release. However, as a registry study from the real world, the necessity of functional evaluation was determined by clinicians according to patients’ clinical condition and the results of angiography. For patients with NSTEMI, it is easier for clinicians to determine the culprit lesions and target vessels based on coronary angiography and clinical context compared to patients with UA and thus may reduce the need for functional assessment. Furthermore, most patients in our study received standard troponin assays rather than high-sensitive troponin (hs-cTn) measurements. Previous studies have demonstrated that the introduction of hs-cTn measurements in place of standard troponin assays can increase the detection of MI (about 4% absolute and 20% relative increases) and reduce the diagnosis of UA in unselected patients with suspected NSTE-ACS (8, 31, 32). Third, the main diseased vessels in this study were the left anterior descending (59.7%), the left circumflex and right coronary artery were only 14.1 and 26.2% respectively. The data in our study may be biased. Previous studies have shown that the lesion of left anterior descending is one of factors leading to the inconsistency between RFR and FFR (28); Fourth, the vasodilator used in this study was adenosine triphosphate, rather than adenosine used in most studies, which may have some impact on the results. However, previous studies have shown that adenosine triphosphate has the same vasodilative effect as adenosine (33); Finally, the sample size included remained small (109 patients, 149 vessels). Therefore, further prospective, multicenter and large-sample clinical studies will be more conducive to guiding the optimal revascularization strategy.

## Conclusion

Resting full-cycle ratio and FFR have good correlation and consistency. The hybrid RFR-FFR strategy highlights considerably enhanced agreement with the FFR-only strategy, whilst making the requirement of vasodilator administration less than a half.

## Data availability statement

The raw data supporting the conclusions of this article will be made available by the authors, without undue reservation.

## Ethics statement

The studies involving human participants were reviewed and approved by The Ethics Committee of Cangzhou Central Hospital, Hebei Medical University attempted to approve the study protocol. The patients/participants provided their written informed consent to participate in this study.

## Author contributions

YL, SZ, ML, and LY provided the conception of the idea for the study, contributed to the development of the methodology, and wrote the manuscript. YL, SZ, ML, and JW analyzed the acquired data. YW, LZ, MC, SZ, and YL were responsible for the interpretation of statistical results. LY revised the manuscript. All authors contributed to the article and approved the submitted version.
